# Management of Refractory Infections in a Neonate With Severe Generalized Epidermolysis Bullosa Simplex: A Case Report

**DOI:** 10.7759/cureus.100404

**Published:** 2025-12-30

**Authors:** Maho Maekawa, Kenji Yoshida, Keiji Tanese, Ayaka Tomita, Akira Ishiko

**Affiliations:** 1 Department of Dermatology, Toho University School of Medicine, Tokyo, JPN; 2 Department of Neonatology, Toho University School of Medicine, Tokyo, JPN

**Keywords:** dowling-meara type, keratin aggregates, krt5 mutation, p.asn176ile mutation, severe generalized epidermolysis bullosa simplex

## Abstract

A female neonate, the second child of non-consanguineous parents without a family history of similar disease, was born with erosions and small blisters on the perioral area, oral cavity, and digits, along with a large blister on the buttocks. Epidermolysis bullosa (EB) was suspected, and she was referred to our hospital on the day of birth. Skin biopsy from the right upper arm revealed histopathological clefting at the dermoepidermal junction, and electron microscopy demonstrated intraepidermal blister formation with keratin aggregates. Genetic analysis confirmed a p.Asn176Ile mutation in the KRT5 gene, and, based on the clinical presentation, a diagnosis of severe generalized EB simplex (EBS) (Dowling-Meara (DM) type) was made. On day 5 of life, she developed fever, and blood culture yielded methicillin-susceptible *Staphylococcus aureus* (MSSA), prompting initiation of antibiotic therapy. Subsequently, she experienced recurrent episodes of bacteremia and fever caused by *Pseudomonas aeruginosa* and *Enterococcus faecalis*.

In this report, we describe the clinical course and management of intractable infections in a neonate with severe generalized EBS harboring a p.Asn176Ile mutation.

## Introduction

Epidermolysis bullosa (EB) is an inherited skin disorder characterized by blistering and erosions of the skin and mucous membranes, induced by minimal mechanical trauma, due to congenital abnormalities in structural proteins involved in adhesion within the epidermis or at the dermoepidermal junction [1]. Based on the level of blister formation and underlying genetic abnormalities, EB is classified into EB simplex (EBS), junctional EB (JEB), dystrophic EB (DEB), and Kindler syndrome [2].

EBS is caused by dysfunction of keratin 5 (KRT5) or keratin 14 (KRT14), which are components of the intermediate filaments in basal keratinocytes, leading to intraepidermal blister formation [3]. In the 2020 international consensus reclassification, EBS was classified into localized (formerly Weber-Cockayne), intermediate (formerly Koebner), and severe generalized (formerly Dowling-Meara (DM)) subtypes, according to clinical severity and genetic background [4]. Severe generalized EBS typically presents with widespread blistering from birth and is often accompanied by palmoplantar keratoderma [4]. In Japan, the estimated number of patients with EBS is 180, of whom approximately 40 have the severe generalized subtype [5,6]. The prognosis during the neonatal period largely depends on infection control, as extensive erosions and disruption of the skin barrier predispose to bacterial, fungal, and viral infections, which can result in sepsis and respiratory failure [4].

In this report, we present a neonate with severe generalized EBS harboring the KRT5 p.Asn176Ile mutation, focusing on the management of recurrent and intractable infections during the clinical course.

## Case presentation

A Japanese female neonate presented with generalized blisters and erosions. She was delivered at 38 weeks and 3 days of gestation via elective cesarean section. Her birth weight was 2945 g, and Apgar scores were 6 at one minute and 9 at five minutes. She was the second child of non-consanguineous parents, with no family history of blistering disorders.

**Figure 1:**
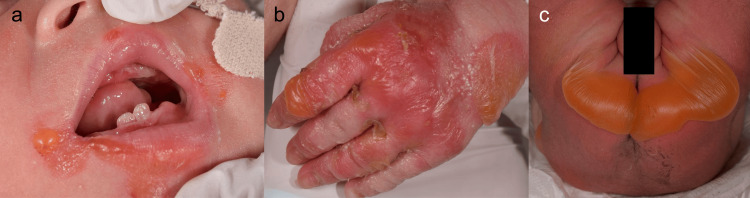
Clinical findings in the patient a) Erosions and blisters up to 10 mm in diameter are observed around the mouth, on the gingival mucosa, and along the mandible. b) Erosions and small vesicles are also present on the fingers, dorsum of the hands, and toes, accompanied by nail plate thickening. c) A blister approximately 100 mm in diameter is present on the buttocks, and erythematous areas with blisters measuring 2 to 10 mm in diameter are also observed on the genital region, both thighs, peri-knee areas, and the right upper arm.

At birth, erosions and blisters up to 10 mm in diameter were observed around the perioral area, gingiva, and mandible. Similar erosions and small blisters were present on the fingers, dorsal hands, and toes. Nail thickening was also noted. A large blister, approximately 100 mm in diameter, was seen on the buttocks, and multiple erythematous blisters, ranging from 2 to 10 mm, were present on the external genitalia, thighs, knees, and right upper arm (Figures 1a-1c).

**Figure 2:**
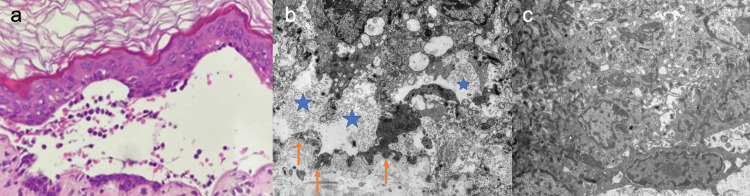
Histopathological findings a) A cleft is observed near the dermoepidermal junction, located within the epidermis while partially preserving the basal layer. b) Intraepidermal blister formation was present. The asterisks indicate the intraepidermal blister cavity, and the arrows indicate the basement membrane. c) Aggregates of keratin are identified within the cytoplasm of epidermal cells, forming the blister roof.

Laboratory evaluation revealed elevated lactate dehydrogenase (LDH) (613 U/L; neonatal reference range: 225-600 U/L) and hypoalbuminemia (2.9 g/dL; neonatal reference range: 3.0-5.4 g/dL), while other findings were unremarkable. A skin biopsy from a blister on the right upper arm demonstrated cleft formation near the dermoepidermal junction, with intraepidermal localization leaving parts of the basal layer intact (Figure 2a). Electron microscopy revealed intraepidermal blistering (Figure 2b), and keratin aggregates were observed within the cytoplasm of blister-roof keratinocytes (Figure 2c).

Genetic analysis was performed using next-generation sequencing of genes associated with EB, with confirmation by Sanger sequencing. This analysis revealed a heterozygous p.Asn176Ile mutation in the KRT5 gene. In addition, a c.7202_7207del variant in the PLEC gene was identified; however, the pathogenic significance of this variant has not yet been reported, and no clinical features suggestive of plectin-associated EB were observed.

Based on the clinical, histopathological, ultrastructural, and genetic findings, a diagnosis of severe generalized EBS (formerly DM type) was established.

**Figure 3:**
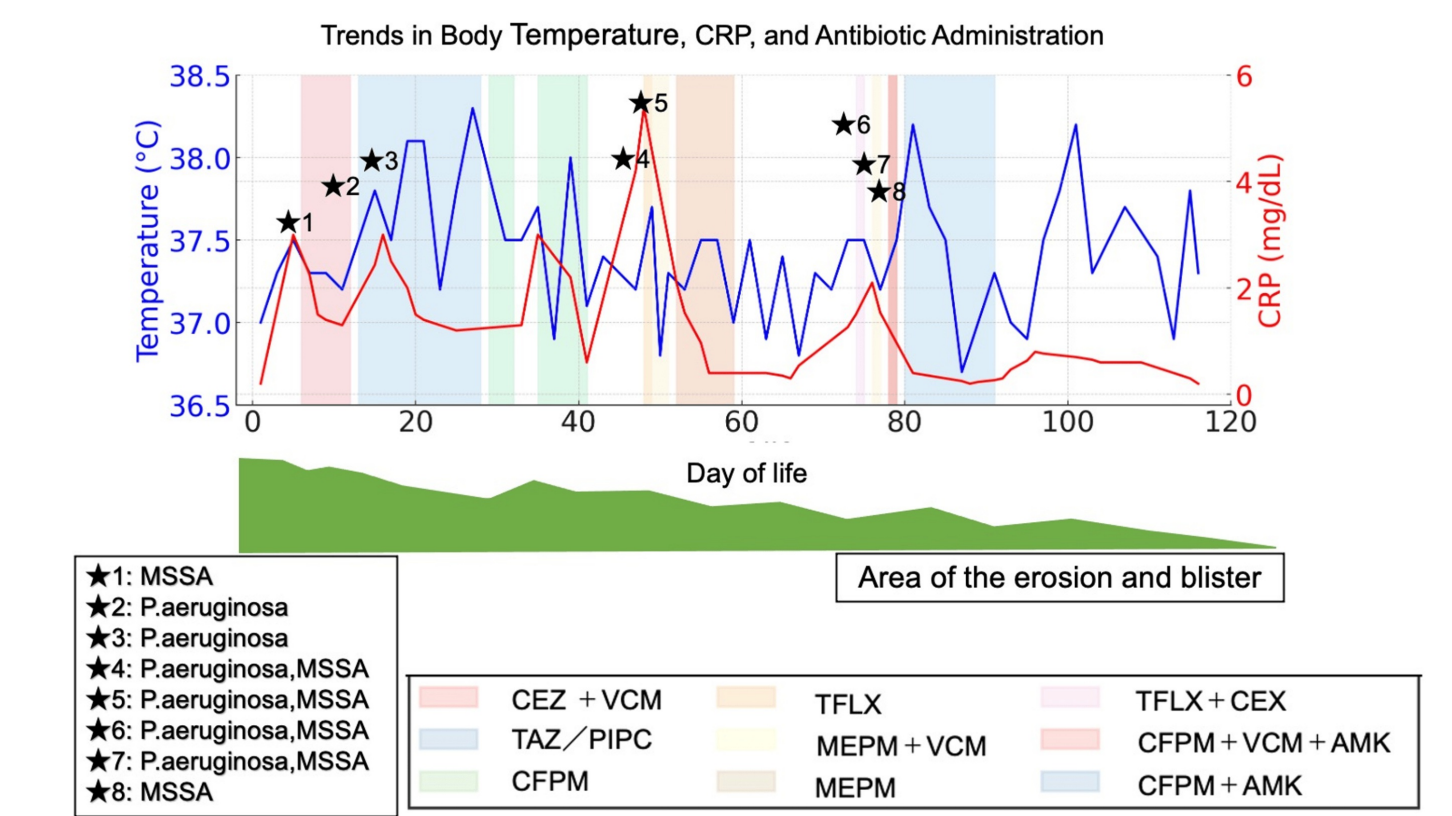
Clinical course, infectious episodes, and antimicrobial therapy On day 5 of life, the patient developed fever, and methicillin-sensitive *Staphylococcus aureus* (MSSA) was isolated from blood cultures. Intravenous cefazolin (CEZ) and vancomycin (VCM) were initiated. On day 8, hoarseness and inspiratory stridor were noted, and a high-flow nasal cannula (HFNC) was applied as needed during episodes of respiratory deterioration. On day 12, *Pseudomonas aeruginosa* was isolated from both blood and wound cultures, prompting a switch to tazobactam/piperacillin (TAZ/PIPC). However, due to the emergence of resistant strains after prolonged administration, the regimen was changed back to cefepime (CFPM) and antimicrobial therapy was continued for a total of 28 days. On day 34, the patient exhibited decreased activity, poor feeding, and a recurrent elevation in C-reactive protein (CRP); CFPM was re-administered from day 35. On day 49, routine blood tests revealed elevated CRP, and blood cultures yielded MSSA and *P. aeruginosa* bacteremia, leading to continuation of CFPM until day 66. Subsequently, on day 74, CRP levels again increased, and blood cultures grew MSSA, *P. aeruginosa*, and *Enterococcus faecalis*. Combination therapy with meropenem (MEPM) and VCM was initiated, resulting in clinical improvement by day 92. Between days 103 and 105, transient fever and malodor of the skin were observed, for which tosufloxacin (TFLX) was administered, with subsequent improvement. Thereafter, CRP normalized, signs of infection resolved, and the area of skin lesions decreased.

On day 5 of life, the patient developed fever, and blood cultures yielded methicillin-susceptible *Staphylococcus aureus* (MSSA). Intravenous cefazolin (CEZ) and vancomycin (VCM) were initiated. Recurrent infections caused by *Pseudomonas aeruginosa* and *Enterococcus faecalis* followed, requiring sequential or combined administration of antibiotics tailored to drug susceptibility, including tazobactam/piperacillin (TAZ/PIPC), cefpirome (CEPM), tosufloxacin (TFLX), meropenem (MEPM), and VCM. Antimicrobial therapy was continued for a total of three months. On day 8, the patient developed mucosal edema and respiratory distress, requiring initiation of high-flow nasal cannula oxygen therapy, which stabilized her respiratory condition (Figure 3).

Skin care consisted of gentle cleansing, deroofing of new blisters, and coverage with custom-fitted wound dressings and topical petroleum jelly, secured with Tubifast® (Mölnlycke Health Care, Göteborg, Sweden). To prevent digital adhesions, each finger was individually protected with Mepitel One® (Mölnlycke Health Care, Göteborg, Sweden).

For hypoalbuminemia, intravenous infusion of 4 g of albumin (1.6 g/kg) was administered on day 11, after which serum albumin remained stable without further supplementation. The skin lesions, particularly in intertriginous areas, demonstrated cycles of blistering, erosion, and re-epithelialization, with a gradual reduction in overall affected surface area. Concurrently, signs of infection resolved, and her general condition improved. At three months of age, she was discharged home in a stable condition. Growth remained slightly below average for her age, but no systemic deterioration was observed thereafter.

## Discussion

EBS is a mechanobullous disorder caused by abnormalities of keratin proteins that constitute the intermediate filaments of basal keratinocytes. Since the basal lamina of the dermoepidermal junction remains intact [7], blistering occurs at a superficial level, and erosions typically re-epithelialize within 7-10 days. Therefore, EBS is generally considered to have a favorable prognosis [7]. However, the severe generalized subtype presents with widespread lesions from birth and carries a significant risk of infection-related mortality [8].

Severe forms of EB are frequently complicated by sepsis secondary to recurrent infections [8]. In a retrospective observational cohort study conducted at a national referral center in Spain, including 214 EB patients (135 DEB, 67 EBS, 8 JEB, and 3 Kindler syndrome), the overall incidence of sepsis was 12.1% [9]. Mortality risk, however, varied substantially by subtype. Fine et al. analyzed 450 patients randomly selected from 3,280 EB cases registered in the United States between September 1986 and April 2002, with biennial longitudinal follow-up. The highest risk of early mortality was observed in JEB, with cumulative mortality exceeding 40% by one year of age, predominantly due to poor growth, sepsis, and respiratory failure. In contrast, other EB subtypes demonstrated lower mortality risks within the first year; nevertheless, severe generalized EBS carried the highest mortality among EBS subtypes, with a cumulative risk of 2.8%. Sepsis was the most common cause of death, observed most frequently in JEB, but also reported in 1.9% of patients with generalized severe EBS.

Taken together, these findings highlight marked differences in infection risk and prognosis among EB subtypes. While JEB is associated with the highest early mortality, severe generalized EBS represents the most severe phenotype within the EBS spectrum, with a disproportionately high risk of infection-related complications compared with other EBS subtypes. Extensive skin erosions from birth, prolonged impairment of the skin barrier, and the need for repeated medical interventions contribute to increased susceptibility to recurrent infections and sepsis in this subgroup.

Accordingly, although the overall mortality rate of severe generalized EBS is lower than that of JEB, careful infection surveillance and aggressive supportive management during the neonatal and early infantile periods are critical. This subtype-specific risk profile underscores the importance of early recognition of disease severity and tailored infection-control strategies, as illustrated by the clinical course of the present case.

Despite widespread blistering and erosions during infancy, the severity of generalized severe EBS tends to diminish with age, and prognosis improves significantly after overcoming neonatal infections. The first six months of life are reported to represent the most vulnerable period for severe infections, while mortality rates decline markedly after one year [10]. These findings underscore that, in patients with severe generalized EBS (EBS-DM), as illustrated in our case, early and appropriate medical interventions are crucial for survival. Equally important is prompt and accurate genetic diagnosis, which not only guides clinical management but also facilitates prognostic assessment.

In the diagnosis of EB, immunofluorescence mapping of basement membrane proteins and electron microscopy are commonly performed, and genetic testing is also considered. Electron microscopy enables precise identification of the level of cleavage by observing the ultrastructural changes at the site of blister formation [11]. In severe generalized EBS, intraepidermal blistering caused by basal keratinocyte cytolysis is accompanied by characteristic keratin aggregates [12].

Because each EB subtype is associated with distinct genetic abnormalities and inheritance patterns, molecular diagnosis is indispensable not only for confirming the clinical diagnosis but also for evaluating the recurrence risk in subsequent generations [11]. 

In our case, electron microscopy demonstrated keratin aggregates within the cytoplasm of blister roof keratinocytes, and genetic testing revealed a p.Asn176Ile mutation in the HIM region of KRT5. Although this represents a previously unreported novel mutation, Asn176 is located within a highly conserved region of the 1A domain, and given that a p.Asn176Ser and a p.Asn175Lys substitution have been reported in association with severe generalized EBS, the p.Asn176Ile variant was considered to be the causative mutation in this case [13,14]. Absence of the mutation in either parent confirmed that the patient was the proband with a de novo pathogenic variant.

Among reported genetic causes of severe generalized EBS, the most frequent variants include KRT14 p.Arg125Cys and p.Arg125His, and KRT5 p.Pro25Leu [14]. These residues are located within the coiled-coil domains essential for filament formation. Mutations disrupt the early stages of filament polymerization, preventing the assembly of stable keratin filaments and resulting in intraepidermal blistering, often observed ultrastructurally as clumped tonofilaments. 

In the present case, a p.Asn176Ile mutation was identified, which is located at the coil 1A-H1 boundary of KRT5, a functional hotspot critical for intermediate filament assembly [15]. Asn176 is required for proper alignment of K5/K14 heterodimers and initiation of higher-order filament formation. Its substitution destabilizes the α-helical structure, impairing dimerization and polymerization. Consequently, the keratin filament network becomes insufficiently organized, rendering basal keratinocyte cytoskeletons fragile and contributing to the pathogenesis of severe EBS. To our knowledge, this is the first documented case of severe generalized EBS associated with a p.Asn176Ile mutation. While additional studies and case accumulation are needed to clarify its pathogenic significance, a previous report described an infant with severe generalized EBS harboring a p.Asn176Ser mutation, who exhibited poor weight gain and recurrent bacterial infections that necessitated intensive care during the first nine months of life [16]. Taken together, these observations suggest that neonates with severe generalized EBS carrying the mutation in Asn176 may present with extensive skin fragility and be susceptible to infectious complications. Therefore, early diagnosis and the establishment of a multidisciplinary management approach - including systemic supportive care, antimicrobial therapy, and daily wound care with dressings - are essential.

## Conclusions

In cases of EB presenting at birth with generalized blistering and erosions, as in the present patient, it is often difficult to establish the precise subtype based on clinical findings alone. In severe generalized EBS, however, early and comprehensive multidisciplinary management can enable patients to overcome initial infectious complications and sepsis, as demonstrated in our case. For definitive diagnosis, immunofluorescence mapping of basement membrane proteins, electron microscopy, and genetic testing are indispensable from the neonatal period.
